# Draft genome sequences of multiple bacterial strains isolated from human feces

**DOI:** 10.1128/mra.00307-24

**Published:** 2024-05-29

**Authors:** Marta Selma-Royo, Liviana Ricci, Davide Golzato, Charlotte Servais, Amir Nabinejad, Federica Armanini, Francesco Asnicar, Federica Pinto, Sabrina Tamburini, Nicola Segata

**Affiliations:** 1 Department CIBIO, University of Trento, Trento, Italy; 2 Department of Experimental Oncology, European Institute of Oncology-IRCCS, Milano, Italy; University of Maryland School of Medicine, Baltimore, Maryland, USA

**Keywords:** human microbiome, unknown species

## Abstract

Bacterial isolation is necessary for functional and mechanistic analyses, and the increased human microbiome diversity revealed by metagenomic sequencing is expanding the relevant cultivation targets. Here, we report 46 draft genome sequences of bacterial isolates obtained from fecal samples of healthy adults in Trento and Milan (Italy), including strains from seven taxonomically uncharacterized species.

## ANNOUNCEMENT

Advances in metagenomics highlighted the hidden diversity of the human gut microbiome (1) attributable to yet-to-be-cultivated bacteria. Here, we report 46 draft genome sequences of bacterial isolates from the CibioCM collection obtained from the stool of nine healthy donors living in Trento (*n* = 7) and Milan (*n* = 2; Italy) in 2022 (Protocol no. 2021–007 by the Ethical Committee of University of Trento).

The single fresh samples (within 1–2 h after collection) were homogenized, serially diluted in phosphate buffer solution, and spread onto several 1.5% agar media adjusted to 6–6.5 pH inside an anaerobic chamber (95% N_2_/ 5% H_2_). The isolation media used were: Yeast Casitone Fatty Acids (2), Brain-Heart Infusion, Gifu anaerobic medium supplemented with 30% rumen fluid, Schaedler media, and Chopped meat medium with carbohydrates. Supplements were added in each independent experiment, including fibers and complex sugars, to target species with the capacity to metabolize them that are of interest due to their health-promoting potential (3). The complete list of supplements is specified in Table 1. Plates were incubated in the anaerobic chamber at 37°C.

**TABLE 1:** Complete list of bacterial isolates, their genomes’ statistics, and details regarding their isolation

Isolate ID	Bacterial species	Accession ID	SRA accession	Genome accession ID	No. of reads (×10^6^)	No. of contigs	Cover.	Assembly Size (bp. ×10^6^)	N50 (bp., 10^5^)	GC content	No. of CDSs	Donor	Isolation media^*a*^
CM229S_58	*Acidaminococcus intestini*	SAMN37976377	SRR26567835	JAWQDR000000000	8.77	64	486	2.26	0.61	49.91	2,096	D22	S
CM18GR_37	*Allisonella histaminiformans*	SAMN37976367	SRR26567838	JAWQEB000000000	3.57	58	247	1.77	0.52	46.38	1,577	D1	GR
CM84Y_56_ID122	*A. histaminiformans*	SAMN37976410	SRR26567807	JAWQCV000000000	0.13	68	8	1.59	0.34	46.4	1,521	D8	YCFA + SB
CM85M_510	*A. histaminiformans*	SAMN37976394	SRR26567806	JAWQCU000000000	10.6	14	812	1.65	3.98	46.19	1,544	D8	M111 + SB
CM74B_57	*Bacteroides fragilis*	SAMN37976406	SRR26567814	JAWQDB000000000	10.8	44	248	5.27	3.56	43.19	4,397	D7	BHI + SB
CM57GR_57_ID722	*Bacteroides uniformis*	SAMN37976364	SRR26567830	JAWQDN000000000	1.66	48	33	4.24	1.42	46.76	3,512	D5	GR
CM18M_72	*Bifidobacterium animalis*	SAMN37976373	SRR26567793	JAWQDW000000000	13.0	18	882	1.92	1.90	60.47	1,552	D1	M2m1
CM18M_75	*Bifidobacterium longum*	SAMN37976374	SRR26567792	JAWQDV000000000	18.3	28	977	2.28	1.45	59.72	1,840	D1	M2m1
CM74B_52	*B. longum*	SAMN37976402	SRR26567819	JAWQDF000000000	25.8	41	1,287	2.48	1.28	59.68	2,078	D7	BHI + SB
CM85Y_71	*Catenibacterium mitsoukai*	SAMN37976399	SRR26567799	JAWQCP000000000	12.0	200	376	2.49	0.21	34.26	2,432	D8	Y3 + SB
CM11M_89	*Catenibacterium tridentinum*	SAMN37976379	SRR26567839	JAWQEC000000000	7.89	112	365	2.45	0.42	34.3	2,439	D1_M	M111
CM21M_65	*C. tridentinum*	SAMN37976378	SRR26567791	JAWQDU000000000	15.6	113	756	2.54	0.45	34.45	2,382	D2_M	M111
CM85M_58	*Clostridium fessum*	SAMN37976396	SRR26567803	JAWQCT000000000	24.5	24	1,005	2.95	3.36	48.21	2,562	D8	M111 + SB
CM85M_61	*Clostridium phoceensis*	SAMN37976398	SRR26567801	JAWQCR000000000	19.2	32	678	3.14	2.86	59.97	2,973	D8	M111 + SB
CM74B_53	*Clostridium sp. CM74B_53*	SAMN37976403	SRR26567818	JAWQDE000000000	1.12	40	40	3.40	1.88	48.93	3,025	D7	BHI
CM84Y_54	*Collinsella sp. CM84Y_54*	SAMN37976407	SRR26567809	JAWQCX000000000	3.11	10	170	2.18	4.67	59.79	1,860	D8	YCFA + SB
CM84B_73	*Coriobacteriaceae sp. CM84B_73*	SAMN37976412	SRR26567810	JAWQCY000000000	18.3	9	1,255	1.83	2.33	65.36	1,599	D8	BHI + SB
CM18GR_53_ID1081	*Dorea formicigenerans*	SAMN37976368	SRR26567827	JAWQEA000000000	7.86	27	392	2.60	2.91	40.92	2,427	D1	GR
CM711B_48	*Dysosmobacter welbionis*	SAMN37976392	SRR26567820	JAWQDG000000000	4.71	23	175	3.38	3.52	59.38	3,217	D7	BHIm
CM710M_42	*Enorma massiliensis*	SAMN37976381	SRR26567825	JAWQDJ000000000	5.04	4	299	2.19	15.32	61.85	1,813	D7	M1
CM710M_44	*Enterocloster aldenensis*	SAMN37976382	SRR26567824	JAWQDI000000000	11.9	64	215	5.84	2.62	50.07	5,268	D7	M1
CM229S_55	*Enterocloster citroniae*	SAMN37976376	SRR26567836	JAWQDS000000000	8.73	33	175	6.34	3.63	49.58	5,752	D22	S
CM57GR_63	*Enterococcus faecalis*	SAMN37976366	SRR26567828	JAWQDL000000000	21.1	27	878	2.95	3.87	37.38	2,794	D5	GR
CM84Y_55	*E. faecalis*	SAMN37976409	SRR26567808	JAWQCW000000000	6.28	26	268	2.90	2.78	37.28	2,758	D8	YCFA + SB
CM93Y_63	*E. faecalis*	SAMN37976389	SRR26567796	JAWQCM000000000	8.36	27	339	2.98	2.49	37.47	2,821	D9	Y
CM74Y_51	*Enterococcus faecium*	SAMN37976401	SRR26567813	JAWZUO000000000	15.6	76	733	2.70	1.02	38.31	2,557	D7	YCFA + SB
CM23Y_61	*Escherichia coli*	SAMN37976386	SRR26567832	JAWQDP000000000	10.4	44	264	5.00	2.95	50.61	4,712	D2	Y
CM57GR_62	*E. coli*	SAMN37976365	SRR26567829	JAWQDM000000000	10.7	80	261	5.12	1.13	50.71	4,782	D5	GR
CM710M_41	*E. coli*	SAMN37976380	SRR26567826	JAWQDK000000000	8.97	86	214	5.22	1.93	50.57	4,874	D7	M1
CM85M_52B	*E. coli*	SAMN37976395	SRR26567804	JAWZUN000000000	9.03	30	225	5.05	3.71	50.52	4,717	D8	M111 + SB
CM93B_62	*E. coli*	SAMN37976388	SRR26567797	JAWQCN000000000	15.1	27	396	4.89	5.43	50.67	4,538	D9	BHIm
CM85Y_73	*Holdemanella biformis*	SAMN37976400	SRR26567798	JAWQCO000000000	7.04	155	300	2.62	0.32	34.27	2,574	D8	Y3 + SB
CM18GR_54	*Oscillospiraceae bacterium CM18GR54*	SAMN37976369	SRR26567816	JAWQDZ000000000	5.39	20	203	2.99	4.82	57.84	2,915	D1	GR
CM18GR_63	*O. bacterium CM18GR63*	SAMN37976372	SRR26567794	JAWQDX000000000	2.40	53	80	3.40	2.31	57.84	3,370	D1	GR
CM710M_45	*O. bacterium CM710M_45*	SAMN37976383	SRR26567823	JAWZUP000000000	4.50	46	153	3.19	3.10	57.55	3,138	D7	M1
CM74B_53B_ID361	*O. bacterium CM74B_53B_ID361*	SAMN37976404	SRR26567817	JAWQDD000000000	0.21	32	10	2.36	1.29	59.21	2,299	D7	BHI
CM84B_55	*Parolsenella catena*	SAMN37976408	SRR26567812	JAWQDA000000000	6.66	8	423	1.81	14.43	65.42	1,562	D8	BHI
CM229S_512_ID1442	*Phascolarctobacterium faecium*	SAMN37976375	SRR26567837	JAWQDT000000000	0.31	8	16	2.19	4.07	43.45	2,099	D22	S
CM22M_65	*Phocaeicola dorei*	SAMN37976384	SRR26567834	JAWQDQ000000000	3.58	80	76	5.73	1.64	42.07	4,732	D2	M110
CM57GR_57_ID721	*P. dorei*	SAMN37976387	SRR26567831	JAWQDO000000000	28.2	37	730	4.98	1.96	41.67	4,027	D5	GR
CM74B_55_ID151	*Slackia isoflavoniconvertens*	SAMN37976405	SRR26567815	JAWQDC000000000	0.16	61	10	2.00	0.49	57.54	1,649	D7	BHI + SB
CM84B_57	*S. isoflavoniconvertens*	SAMN37976411	SRR26567811	JAWQCZ000000000	2.28	71	55	2.20	0.99	57.57	1,826	D8	BHI + SB
CM85M_59	*Veillonella atypica*	SAMN37976397	SRR26567802	JAWQCS000000000	10.3	28	616	2.13	1.71	38.98	1,910	D8	M111 + SB
CM18GR_62_ID1101	*Veillonella parvula*	SAMN37976371	SRR26567795	JAWQDY000000000	28.2	4	1,775	2.07	6.42	38.61	1,832	D1	GR
CM711B_41	*V. parvula*	SAMN37976390	SRR26567822	JAWQDH000000000	5.49	10	332	2.10	5.22	38.6	1,849	D7	BHIm
CM85Y_47	*V. parvula*	SAMN37976393	SRR26567800	JAWQCQ000000000	11.8	7	707	2.06	6.29	38.68	1,840	D8	Y3 + SB

^*a*^ Isolation media: GR: Gifu media [+ Rumen Fluid 30%, (vol/vol)]; YCFA: Yeast Casitone Fatty Acids; Y: Yeast Casitone Fatty Acids + Sucrose (2 g/L), Cellobiose (2 g/L); Y3: Yeast Casitone Fatty Acids (without glucose) + Sucrose (2 g/L), Maltose (2 g/L), Pectin (2g/L), Arabinoxylan (2 g/L); M2m1: Yeast Casitone Fatty Acids (without short-chain fatty acids and vitamin solutions) + Rumen fluid [30% (vol/vol)], Glucose (1 g/L), Soluble starch (2 g/L), Cellobiose (2 g/L), Pectin (2 g/L), Arabinoxylan (2 g/L); M110: Chopped meat media with carbohydrates; M111: Chopped meat media with carbohydrates + Pectin (2 g/L), Arabinoxylan (2 g/L); M1: Chopped meat media with carbohydrates + Ciprofloxacin, Streptomycin, Gentamycin, and Neomycin; S: Schaedler media; BHI: Brain-Heart Infusion; BHIm: Brain-Heart Infusion + Yeast extract (2.5 g/L), Cellobiose (2 g/L), Maltose (2 g/L); SB: Sheep Blood [4%, (vol/vol)].

After 5 days, a single colony was inoculated in the correspondent liquid media. From 48 h to 72 h liquid cultures, genomic DNA was isolated using the Wizard Genomic DNA Purification Kit (Promega), and sequencing libraries were prepared with Illumina DNA prep and Tagmentation kit (Illumina) following the recommended protocol. Libraries were sequenced (150 bp paired-ends reads) on a NovaSeq6000 instrument with S4 flowcell reagents (Illumina) at the University of Trento (Italy), after a cleaning step with 0.6× Agencourt AMPure XP beads.

Raw reads were pre-processed with Trim Galore to remove low-quality and adapter-contaminated reads (parameters: “--stringency 5—length 75—quality 20—max_n 2 --trim-n”; https://github.com/FelixKrueger/TrimGalore). Illumina PhiX control sequences and human DNA reads were removed using Bowtie2 (4) against the corresponding reference genomes. Genome assembly was performed using SPAdes 3.15.2 (parameters: --careful -k 21,33,55,77,99,127 --only-assembler) (5) removing contigs shorter than 1,000 bp. QUAST v5.1.0rc1 (6) was used to calculate assembly statistics. Completeness and contamination of the assemblies were assessed by CheckM v1.1.2 (*lineage_wf workflow*) (7), while median coverage was obtained with CMseq v1.0.4 (https://github.com/SegataLab/cmseq). Genomes showing >85% completeness and no contamination were included. Taxonomic assignment was performed using Phylophlan metagenomic v.3.0.36 with the database SGB.Jun23 (8). Default parameters were used for all software unless otherwise specified.

Among the isolated strains, we identified seven previously undescribed taxa belonging to the Oscillospiraceae family and *Clostridium* genus (Table 1). Other bacteria with low representation in the databases are also present in the collection, such as *E. massiliensis* and *Dysosmobacter wellbionis*. The potential association of some of these taxa with host health was described (9), supporting the relevance of the CibioCM bacterial collection in future studies.

## Data Availability

This study is available under NCBI BioProject ID PRJNA939950. The reads and the assembly for the genomes are available under accession reported in Table 1.
